# Oxidative Stress- and Autophagy-Inducing Effects of PSI-LHCI from *Botryococcus braunii* in Breast Cancer Cells

**DOI:** 10.3390/biotech11020009

**Published:** 2022-03-31

**Authors:** Freisa M. Joaquín-Ovalle, Grace Guihurt, Vanessa Barcelo-Bovea, Andraous Hani-Saba, Nicole C. Fontanet-Gómez, Josell Ramirez-Paz, Yasuhiro Kashino, Zally Torres-Martinez, Katerina Doble-Cacho, Louis J. Delinois, Yamixa Delgado, Kai Griebenow

**Affiliations:** 1 Department of Chemistry, University of Puerto Rico, Río Piedras Campus, San Juan 00925, Puerto Rico; freisa.joaquinovalle@upr.edu (F.M.J.-O.); gracegpr@gmail.com (G.G.); vanessabarcelo1@gmail.com (V.B.-B.); andraous.saba@upr.edu (A.H.-S.); nicole.fontanet@upr.edu (N.C.F.-G.); josellyaima@hotmail.com (J.R.-P.); zallytorres@gmail.com (Z.T.-M.); katerina.doble@edu.uag.mx (K.D.-C.); delinoisjeanlouis@gmail.com (L.J.D.); 2 Graduate School of Science, University of Hyogo, Kobe 678-1297, Japan; kashino@sci.u-hyogo.ac.jp; 3 Biochemistry & Pharmacology Department, San Juan Bautista School of Medicine, Caguas 00725, Puerto Rico

**Keywords:** microalgae, *Botryococcus braunii*, purification, photosystem I, light-harvesting complex I, reactive oxygen species, cancer, cell viability, necrosis, autophagy

## Abstract

*Botryococcus braunii* (*B. braunii*) is a green microalga primarily found in freshwater, reservoirs, and ponds. Photosynthetic pigments from algae have shown many bioactive molecules with therapeutic potential. Herein, we report the purification, characterization, and anticancer properties of photosystem I light-harvesting complex I (PSI-LHCI) from the green microalga *B. braunii* UTEX2441. The pigment–protein complex was purified by sucrose density gradient and characterized by its distinctive peaks using absorption, low-temperature (77 K) fluorescence, and circular dichroism (CD) spectroscopic analyses. Protein complexes were resolved by blue native-PAGE and two-dimensional SDS-PAGE. Triple-negative breast cancer MDA-MB-231 cells were incubated with PSI-LHCI for all of our experiments. Cell viability was assessed, revealing a significant reduction in a time- and concentration-dependent manner. We confirmed the internalization of PSI-LHCI within the cytoplasm and nucleus after 12 h of incubation. Cell death mechanism by oxidative stress was confirmed by the production of reactive oxygen species (ROS) and specifically superoxide. Furthermore, we monitored autophagic flux, apoptotic and necrotic features after treatment with PSI-LHCI. Treated MDA-MB-231 cells showed positive autophagy signals in the cytoplasm and nucleus, and necrotic morphology by the permeabilization of the cell membrane. Our findings demonstrated for the first time the cytotoxic properties of *B. braunii* PSI-LHCI by the induction of ROS and autophagy in breast cancer cells.

## 1. Introduction

Microalgae generate novel products for the food, agriculture, cosmetic, pharmaceutical, and biofuel industries [1]. Microalgal components include marketable high-value bioactive compounds, such as pigments (chlorophyll, carotenoids, β-carotene, phycobiliproteins, xanthophylls), vitamins, and antioxidants [2]. Among the different types of photosynthetic pigments produced by microalgae, chlorophyll *a* and its metabolites have strong antioxidant properties [3]. Chlorophyll, as an essential bioactive compound with health-boosting effects in humans, is regularly used in the pharmaceutical industry due to its antimutagenic activity in the form of the soluble derivative chlorophyllin [4]. The biological properties as an anticancer molecule are presumably attributed to the free radical scavenging ability of the porphyrin ring or by binding to carcinogens [4,5].

Microalgal biomedical research studies and reviews have highlighted carotenoid and chlorophyll pigments in vitro antiproliferative and anticancer activity against various cancer cell lines [6,7,8,9]. Moreover, microalgal protein and biomedical properties of peptides have also been explored [10]. However, reports on the anticancer properties of pigment–protein complexes are limited to higher plants. Saha et al. [11] evaluated *Spinacia oleracea* ROS-mediated photosystem I (PSI) anticancer activities against human lung carcinoma (A549) and mouse melanoma (B16F10) cell lines. The authors reported that PSI induced apoptosis of cancer cells via a mitochondria-dependent internal pathway accompanied by activation of caspase-3. These findings suggest that protein complexes from photosynthetic organisms, including green microalgae, possess potential as agents for cancer therapy.

Algal photosynthetic energy conversion is driven by large multisubunit pigment–protein complexes located in the photosynthetic membrane, namely, PSI and PSII, which operate in series, each with its own polypeptide rich reaction center and antenna pigments light-harvesting complexes (LHCI, LHCII) associated with them [12,13,14,15,16]. PSI is the leading generation site of ROS owed to the culmination of electron transfer reactions in the stromal thylakoid membranes and recovered mainly as a trimer or a monomer from cyanobacteria and as a monomer or supercomplex of a monomer with the associated light-harvesting antenna complex of PSI-LHCI in higher plants and green algae [17,18,19].

*Botryococcus braunii* (*B. braunii*) UTEX2441 is a green colonial microalga, highly studied due to its hydrocarbon synthesis, secretion, and accumulation capability, owing to a lipid-containing matrix, making it a promising biofuel production source, however, with some limitations [20,21,22,23]. A recent study by van den Berg et al. [24] on *B. braunii* light-harvesting pigment complexes (LHCs) purification reported a monomeric and a less stable trimeric fraction with a chlorophyll pigment composition comparable to LHCII in plants and algae. In contrast, a carotenoid composition variability was observed [24,25]. *B. braunii* studies have mainly focused on this microalga’s hydrocarbon-accumulation properties, overlooking their full research potential. Investigating *B. braunii* PSI-LHCI in the cancer therapeutics context provides an added value to the use of this microalga biomass. During initial screening tests looking for in vitro susceptibility of cancer cell lines (HeLa cervical carcinoma, MDA-MB-468 breast adenocarcinoma, and MDA-MB-231 breast adenocarcinoma) to PSI-LHCI, MDA-MB-231 cells showed the best response, the data were consistent and reproducible. This cell line was subsequently selected and used further on.

Here, we present the first study on the isolation, characterization, and biological properties of PSI-LHCI from *B. braunii* and its potential biomedical applications, such as cancer treatment. *B. braunii* PSI-LHCI was isolated by sucrose density gradient ultracentrifugation and thoroughly characterized to include the contribution from LHCI. Thus, our absorption maxima (679 nm) differs from the Saha et al. study (675 nm) [11]. Moreover, in a ROS-dependent manner, PSI-LHCI cytotoxicity was demonstrated in the incurable and fatal triple-negative breast cancer (TNBC) MDA-MB-231 cell line by necrotic and autophagic cell death pathways, in contrast to apoptotic cell death from PSI isolated from *Spinacia oleracea*, presumably due to variations arising among different organisms.

## 2. Materials and Methods

### 2.1. Culture Strain and Growth Conditions

*B. braunii* UTEX 2441 strain race A was obtained from The Culture Collection of Algae at the University of Texas at Austin. Cells were grown at 23 °C in modified Chu 13 medium in a UTEX 2L photobioreactor system, which provided gas exchange and mixing by air bubbling [22]. Cultures were under 50 μmol photons m^−2^s^−1^ of white light illumination (Philips, Amsterdam, Netherlands) with a 16:8 h light–dark cycle. *B. braunii* cells from 50 mL suspensions were harvested at their logarithmic growth phase by centrifugation at 3000× *g* at 4 °C for 10 min and washed twice in breaking buffer A (50 mM HEPES (pH 7.2), 5 mM MgCl_2_, 5 mM CaCl_2_) with 20% glycerol (*v*/*v*).

### 2.2. Thylakoid Membrane Preparation

All preparations were performed in the dark and at 4 °C. *B. braunii* cells were suspended in ice-cold breaking buffer A and supplemented with 1 mM EDTA and a 1× protease inhibitor cocktail (Sigma-Aldrich, St. Louis, MO, USA). Cells were disrupted in a 15 mL chamber of a Mini-BeadBeater homogenizer (BioSpec Products, Bartlesville, OK, USA) filled halfway with ice-cold 0.5 mm glass beads for 25 cycles of 15 s beating and 2 min of cooling. Cell debris, intact cells, starch granules, and glass beads settled down as a pellet and were removed by centrifugation at 3000× *g* at 4 °C for 5 min. The resulting supernatant was collected and centrifuged at 32,600 rpm with a Thermo Scientific AH-650 rotor at 4 °C for 30 min. The pellet of thylakoid membranes was suspended in the supplemented breaking buffer A containing 1% (*w*/*v*) *n*-dodecyl β-d-maltoside (DDM) and solubilized under gentle stirring for 15 min. Subsequently, the solubilized thylakoids were centrifuged at 36,200 rpm with a Thermo Scientific AH-650 rotor at 4 °C for 20 min to obtain stroma membranes as a supernatant; the resulting pellet corresponded to the grana preparation and was discarded. The stromal thylakoid supernatant was stored at −80 °C until use.

### 2.3. Purification of PSI-LHCI from Botryococcus braunii

Stromal thylakoids were loaded onto a discontinuous sucrose density gradient in the range of 0.1–1.5 M in supplemented breaking buffer A with 1 mM EDTA, 1× protease inhibitor cocktail, 0.03% (*w*/*v*) DDM, and centrifuged at 37,500 rpm with a Thermo Scientific TH-641 rotor at 4 °C for 16.5 h. The lower green band obtained, corresponding to the PSI-enriched fraction, was collected using a syringe, concentrated with a centrifugal filter unit (10,000 NMWL, Millipore, Burlington, MA, USA), and stored at −80 °C.

### 2.4. Spectroscopic Studies

Absorption spectra were measured at room temperature (RT) using a UV-2450 UV/Vis spectrophotometer (Shimadzu, Kyoto, Japan) using 1 cm (10 mm) path length quartz cuvettes; scanning was performed on the visible region up to 800 nm. PSI-LHCI sucrose and glycerol fractions low-temperature 77 K fluorescence emission spectra were recorded from 630 to 800 nm and measured using a FluoroMax-4 spectrofluorometer (Horiba Scientific, Kyoto, Japan) equipped with a low-temperature holder at an excitation wavelength of 436 nm. Before 77 K fluorescence measurements, each sample was cooled in liquid nitrogen in a cryostat. Pigment content was determined after extraction in 90% (*v*/*v*) methanol according to Meeks and Castenholz extinction coefficient and Lichtenthaler and Buschmann equations [26,27]. Protein concentration was determined with the BCA protein assay kit (Pierce, Waltham, MA, USA), using diluted BSA standards from a 2 mg/mL stock. Circular dichroism (CD) spectra were obtained using a JASCO J-1500 CD spectrometer at a scanning speed of 50 nm/min, a bandwidth of 1 nm, and at 25 °C. Visible range measurements between 350 and 750 nm were recorded with a 10 mm quartz cuvette with previously desalted sucrose gradient purified PSI-LHCI and thylakoid membrane samples at 0.4 mg/mL Chl concentration. Final spectra were normalized to the maximum peak in the red region, averaged from three scans, and corrected by subtracting the breaking buffer A spectra acquired under the same conditions.

### 2.5. Protein Composition Analysis

The protein composition of *B. braunii* was analyzed using SDS-PAGE with a 10% (*w*/*v*) SDS polyacrylamide resolving gel and 5% SDS polyacrylamide stacking gel and run according to the manufacturer’s protocol (Bio-Rad, Hercules, CA, USA) with a 1× Tris/Glycine/SDS buffer system on a Mini-Protean electrophoresis cell system (Bio-Rad, Hercules, CA, USA). Beforehand, protein samples were solubilized in a Laemmli sample buffer (2×) containing 50 μL of β-mercaptoethanol and incubated at 95 °C for 5 min in a gradient thermal cycler (Eppendorf, Hamburg, Germany) immediately before electrophoresis. A prestained protein standard (Bio-Rad, Hercules, CA, USA) was used as a molecular weight marker to follow the progress of proteins 5–10 mm into the resolving part of the gel. Identification of protein subunits submitted as gel plugs was performed by running samples for 1 h on a Dionex LC system and Orbitrap mass spectrometer for LC–MS/MS (Proteomics Facility, Austin, TX, USA). Blue NativePAGE (BN-PAGE) of *B. braunii* thylakoids and the sucrose gradient purified PSI-LHCI sample (0.7 mg/mL Chl) was performed in a 4–16% gradient Novex Bis-Tris gel, 5% G-250 sample additive, pre-chilled 1× running buffers, and a 1× NativePAGE sample buffer, at constant 150 V and for up to 120 min, as indicated in the manufacturer’s recommended protocol (Invitrogen, Waltham, MA, USA). Mass estimation was achieved using NativeMark unstained protein standard (Invitrogen, Waltham, MA, USA) to generate a molecular weight (MW) standard curve of the log MW versus the migration distance of the protein through the gel divided by the migration distance of the dye front (R_f_). Protein bands were detected with Coomassie Brilliant Blue G-250 and a silver staining kit (Sigma-Aldrich, St. Louis, MO, USA). Two-dimensional (2D) native/SDS-PAGE was accomplished in a gradient 4–12% NuPAGE Bis-Tris gel in 1× MES running buffer at a constant 150 V for 1 h with the excised PSI-LHCI BN-PAGE gel lane obtained that underwent reduction, alkylation, and quenching as indicated in the manufacturer’s protocol (Invitrogen, Waltham, MA, USA). We used a prestained protein standard (Thermo Fisher Scientific, Waltham, MA, USA) to estimate the PSI components’ apparent size.

### 2.6. Cell Viability Assay

MTS assay was performed using the CellTiter 96^®^ AQ_ueous_ Non-Radioactive Cell Proliferation Assay (Promega G5421) to determine the number of viable MDA-MB-231 cells after treatment with PSI-LHCI. MDA-MB-231 cells were obtained from the American Type Culture Collection (Manassas, VA, USA) and maintained in L-15 medium supplemented with l-glutamine, 10% FBS, and 1% Pen/Strep. Cells were seeded in 96-well plates at a density of 10,000 cells/well and incubated for 24 h at 37 °C in a 0% CO_2_ atmosphere. After 24 h of incubation, cell arrest was achieved by reducing FBS concentration in the medium to 1% for 18 h. Next, cells were washed with 1× PBS and treated with different concentrations (6.25, 12.5, 25, 50 µg/mL) of PSI-LHCI for 12 and 24 h. At the end of treatment, cell viability was determined by the MTS assay as described by the manufacturer. Firstly, 20 µL of the MTS assay solution was added to each sample well and incubated for 1 to 4 h followed by absorbance measurements recorded at 490 nm using a 96-well Tecan reader. Staurosporine (2 µM) was used as a positive control, and the buffer extraction medium and untreated cells were used as negative controls. The IC_50_ value was calculated from the MTS assay results. The cell viability percentiles obtained were analyzed through the GraphPad Prism 6 software program. At least three experimental replicate results were performed.

### 2.7. Cellular Internalization Study of PSI-LHCI

Cells were plated in 4-well plates at a density of 10,000 cells/well and incubated overnight in L-15 medium. Next, cells were arrested for 18 h and incubated with PSI-LHCI (12.5 µg/mL) for 6 and 12 h. Afterward, cells were washed three times with PBS 1× for 10 min, stained with CellMask™ green plasma membrane dye (Ex/Em = 522/535 nm) for 15 min, and fixed for 20 min with a 3.7% formaldehyde solution at room (RT). After removing the fixing solution and washing it three times with PBS 1× for 5 min, cells were incubated with the nuclear counterstain DAPI. Cells were analyzed by in-depth optical sectioning through the sample along the *z*-axis (Z-series), generating a 3D image in a Nikon Eclipse Ti confocal laser scanning microscope with a 60× objective and the appropriate dye channels. Untreated control cells were used to detect autofluorescence. Fluorescence intensity was analyzed using NIS-Elements Advanced Research Imaging Software (version 5.20).

### 2.8. ROS/Superoxide Production Assay

The ROS/Superoxide Detection Assay Kit (Abcam ab139476) was used to detect total ROS and superoxide production in live MDA-MB-231 cells. First, cells were seeded in 4-well plates at a density of 10,000 cells/well and incubated for 24 h in L-15 medium containing l-glutamine, 10% FBS, and 1% penicillin/streptomycin at 37 °C in 0% CO_2_. After 18 h of serum starvation in 1% FBS L-15 medium, cells were treated with PSI-LHCI (12.5 µg/mL) for 1 or 24 h. Before induction, cells were pretreated for 30 min with 5 mM of the negative control and ROS inhibitor (*N*-acetyl-l-cysteine). Next, cells were loaded with 2× ROS/Superoxide detection mix, prepared by mixing the green oxidative stress reagent (5 mM) and the orange detection reagent (5 mM) in 1× wash buffer. A 1:2500 diluted staining solution containing PSI-LHCI and the ROS inducer pyocyanin (200 µM) were obtained. After incubation for 30 min to 1 h at 37 °C, the staining mix solution was removed, washed twice with 1× wash buffer, and the cells were examined using a Nikon Eclipse Ti confocal laser scanning microscope with filter sets compatible with fluorescein (Ex/Em = 490/525 nm) and rhodamine (Ex/Em = 550/620 nm). Fluorescence intensity was analyzed using NIS-Elements Advanced Research Imaging Software (version 5.20).

### 2.9. Annexin V/Propidium Iodide (Apoptosis/Necrosis) Assay

Cell death was evaluated using propidium iodide (PI) and Alexa Fluor^®^ 488 annexin V/Dead Cell Apoptosis kit (Invitrogen V13241), and the DNA-selective and membrane-impermeant fluorescent dye Nuclear Green™ DCS1 (AAT Bioquest 17550). Briefly, MDA-MB-231 cells were seeded in 4-well plates and incubated for 24 h in L-15 medium supplemented with l-glutamine, 10% FBS, and 1% Pen/Strep at 37 °C in 0% CO_2_. After 18 h serum starvation in 1% FBS L-15 medium, cells were treated with PSI-LHCI (12.5 µg/mL) and incubated at 37 °C for 24 h. Afterward, cells were washed with 1× PBS and incubated with DAPI, 5 µL Annexin V, and PI (75 µM) for 5 min in the dark or were incubated with Nuclear Green™ DCS1 for 15 min. Cells were fixed with 3.7% formaldehyde solution for 20 min, washed three times with 1× PBS, and covered with glycerol before visualization. Fixed cells were analyzed to confirm cell death under a Nikon Eclipse Ti confocal laser scanning microscope utilizing a 20× or 60× oil objective with filter sets compatible with DAPI (Ex/Em = 405/420–480 nm), Annexin V Alexa Fluor excited at 488 nm with no emission detected, PSI-LHCI (Ex/Em = 640/663–738 nm, PI (Ex/Em = 561/600–674 nm), and Nuclear Green™ DCS1 (Ex/Em = 503/526 nm).

### 2.10. Autophagy Assay

Autophagy was performed in MDA-MB-231 cells using the Autophagy Assay Kit (Abcam ab139484) according to the manufacturer’s protocol. Briefly, cells were seeded in 4-well plates at a density of 10,000 cells/well containing L-15 medium supplemented with l-glutamine, 10% FBS, and 1% Pen/Strep at 37 °C in 0% CO_2_. The autophagy inducer rapamycin (500 nM) and the lysosomal activity inhibitor chloroquine (20 µM) were used as positive controls; untreated cells in media and extraction buffer were used as negative controls. After 24 h of initial seeding, cells were arrested for 18 h and incubated for 24 h with PSI-LHCI (12.5 µg/mL) in 1% FBS L-15 media. After treatment, cells were washed twice with 1× assay buffer containing 5% FBS and stained using 500 µL of the Green Microscopy Dual Detection Reagent for 30 min in the dark at 37 °C, and 0% CO_2_. Next, cells were washed with 100 µL of 1× assay buffer without FBS and fixed with 3.7% formaldehyde solution for 20 min at RT and subsequently washed three times with 1× assay buffer without FBS. The autophagic vacuole fluorescence signal was analyzed by confocal microscopy using a standard FITC (green) filter set. Nuclei were counterstained with DAPI (blue).

### 2.11. Statistical Analysis

All experiments were performed (at least) as three biological replicates, plotting values with an average of eight measurements for each treatment condition as mean ± SD. The quantitative data were analyzed with the statistical software GraphPad Prism 6. Statistical analysis was performed using a two-way analysis of variance (ANOVA).

## 3. Results and Discussion

### 3.1. Thylakoid Membrane Preparation and Solubilization

The thylakoid membranes of *B. braunii* were solubilized after a preliminary extraction process to evaluate the optimum DDM concentration was performed. Mild non-ionic detergents, such as DDM, were widely used to solubilize intact and functional photosynthetic complexes from membranes and maintain the proteins in the solution [28]. We found that the optimal solubilizing condition for the extraction of PSI from stroma thylakoid membranes (STM) was 1% (*w*/*v*) DDM, confirmed by the high absorbance peak at 664 nm at that concentration. A minor red shift of 6 nm was observed in 1% (*w*/*v*) DDM, the PSI-rich STM sample, denoting contribution from light-harvesting complex proteins, free pigments, and PSI in the preparation [29,30,31].

### 3.2. PSI-LHCI Complex Purification and Spectroscopic Characterization

**Figure 1:**
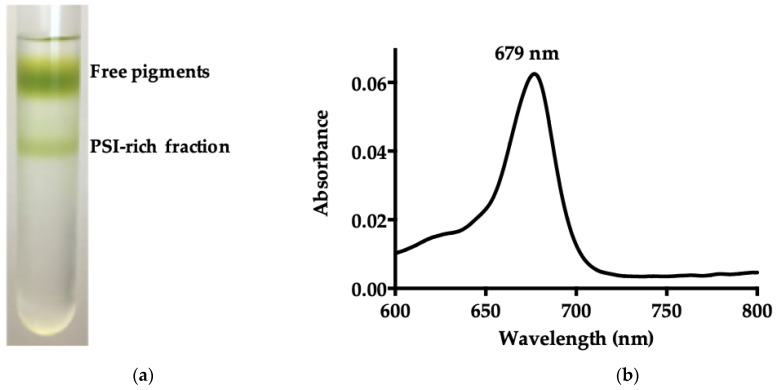

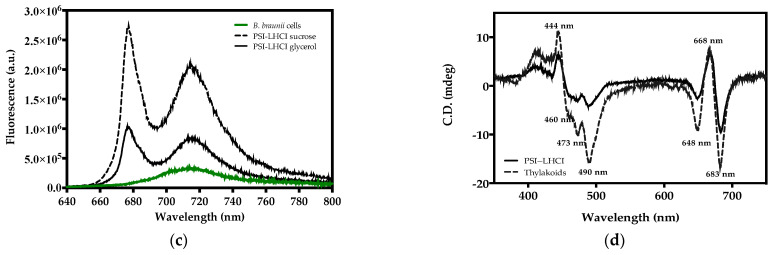
Purification and characterization of PSI-LHCI of *B. braunii*. (**a**) Sucrose gradient separation of *B. braunii* STM. The upper band consists of free pigments and unbound proteins, and the lower green band corresponds to the PSI-rich fraction; (**b**) room temperature absorption spectrum of the PSI-LHCI complex sucrose gradient fraction; (**c**) 77 K fluorescence emission spectra of the PSI-LHCI sucrose gradient fraction (PSI-LHCI sucrose) and the 5–30% glycerol gradient fraction (PSI-LHCI glycerol), and *B. braunii* cells, excitation wavelength 436 nm; (**d**) visible CD spectra of purified PSI-LHCI complex and thylakoid membranes measured in a 10 mm quartz cuvette, at a scanning speed of 50 nm/min, a bandwidth of 1 nm, and 25 °C. Sample concentration was adjusted to 0.4 mg/mL Chl and the spectra normalized at the maximum in the Qy region.

*B. braunii* PSI-LHCI was purified by loading the DDM-solubilized STM onto a 0.1–1.5 M sucrose density gradient. Figure 1a presents the two distinct sucrose density gradient bands obtained, with the lowermost green band corresponding to the PSI-rich fraction. The freshly purified fraction was pooled, concentrated, and an absorption spectrum at room temperature of a PSI-LHCI supercomplex was obtained (Figure 1b), which showed a maximum peak at 679 nm [32,33].

The low temperature (77 K) fluorescence emission spectra of *B. braunii* cells and PSI-LHCI from 0.1–1.5 M sucrose and 5–30% glycerol gradient fractions were obtained using 436 nm excitation. *B. braunii* cells presented a broad emission band at ~715 nm (Figure 1c). PSI-LHCI sucrose and glycerol gradient fractions revealed an additional narrower peak exhibiting a maximum at ~677 nm. The peak detected at ~677 nm occurs as a consequence of the detachment of LHCI from PSI and the inability to effectively transfer its excitation energy, following cell disruption and thylakoid membrane preparation, solubilization, and purification in the presence of 0.03% (*w*/*v*) DDM. If the photosystems remain connected with the LHC antenna, this fluorescence emission peak is absent, as seen in the control (*B. braunii* cells) [34]. The 77 K emission maxima at ~715 nm are characteristically observed in PSI [35]. At 77 K temperatures, photosynthetic reactions cease, and valuable information regarding the organization of the photosynthetic apparatus can be obtained, such as the connection of photosystems with light-harvesting complexes, as reflected on the intensity and fluorescence peaks observed [36].

Visible CD spectroscopy revealed the characteristic pigment–pigment and pigment–protein organization of the *B. braunii* PSI core complex with the antenna system LHCI (PSI-LHCI) and thylakoids (Figure 1d) [37]. The positive peak at 668 nm and negative peak at 683 nm corresponds to chlorophyll dimers resulting from the excitonic interplay of Chl a in PSI-LHCI supercomplexes, whereas the negative peak at 648 nm is attributed to Chl b [38,39,40]. From the Soret region analysis, the PSI-LHCI visible spectrum showed a positive peak at 444 nm originating from Chl a, and three negative peaks at 460, 473, and 490 nm prominently observed in thylakoids due to Chl b and arising from LHCII, respectively [38,40,41,42]. The CD spectra of PSI-LHCI and *B. braunii* thylakoids, although similar, presented a slight shift and peaks with somewhat different intensities as explained by the difference in pigment contribution and content from the samples (Table 1). These results are comparable to those reported in the Qy region of the CD spectrum of PSI-LHCI in the green alga *Chlamydomonas reinhardtii* (*C. reinhardtii*). This indicated the presence of LHCI in the sucrose gradient purified fraction of PSI and confirmed the absence of any significant contribution from LHCII or PSII [43].

In Table 1, the analysis of the pigment content of *B. braunii* cells, thylakoid membrane, and the PSI-LHCI complex revealed substantial differences amongst them. A strong predominance of Chl a is observed in all samples, particularly for PSI-LHCI with (1.50 μg/mL) compared to Chl b (0.45 μg/mL). For PSI-LHCI, the Chl a/b ratio obtained was 3.4, a much lower value if compared to higher plants, such as *Arabidopsis thaliana* (9.7), and the ratio observed in the green microalga *C. reinhardtii* (4.4) [33,44]. A possible increase in abundance in the peripheral pigment antenna system may contribute to these results [45]. The purification treatment influenced the spectroscopic features of PSI-LHCI. Compared to the preparation by glycerol gradient centrifugation, the sucrose fraction displayed the characteristic absorption spectra, fluorescence emission, and CD signals validating PSI-LHCI identity and was subsequently selected as the main purification method.

### 3.3. PSI-LHCI Polypeptide Composition Analysis by SDS-PAGE and BN-PAGE/2D-PAGE

**Figure 2:**
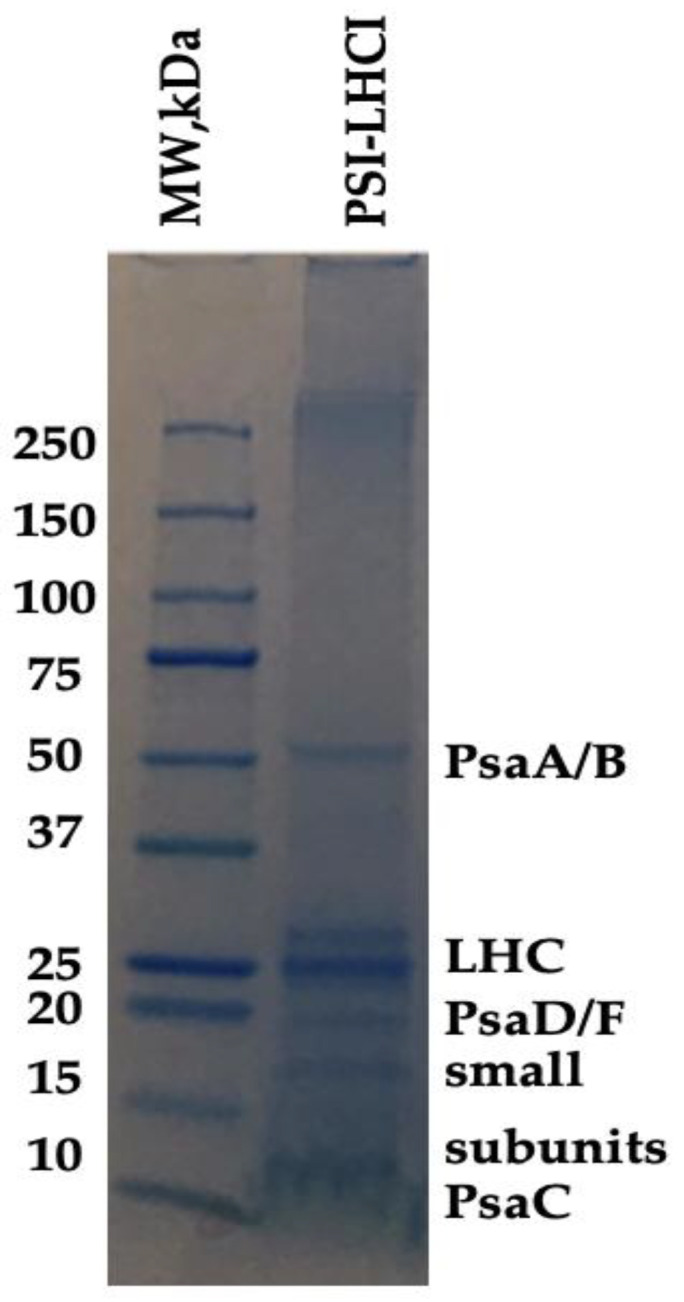
Protein composition of *B. braunii* PSI-LHCI. Polypeptide composition of *B. braunii* PSI-LHCI (1.0 mg/mL) identified by LC–MS/MS. Electrophoresis was performed on a 4–20% polyacrylamide gel. The gel was Coomassie blue-stained.

SDS-PAGE evaluated the polypeptide composition of *B. braunii* PSI-LHCI. As observed in Figure 2, our results indicate the presence of six bands, four of them being small subunits detected in the region of 9–30 kDa. A diffuse band was detected with an apparent molecular weight of ~62 kDa corresponding to PSI reaction center core subunits; Photosystem I P700 chlorophyll *a* apoprotein A1 (PsaA) and Photosystem I P700 chlorophyll *a* apoprotein A2 (PsaB), validated quantitatively by LC–MS/MS for the identification of *B. braunii* PSI-LHCI proteins (Table 2). The bands visualized in the 20–30 kDa range were assigned to the LHC antenna and PSI subunits PsaD and PsaF. In contrast, those that appear around 10 kDa arose from small PSI subunits, such as a PSI iron–sulfur center subunit VII and PsaC [33,46]. The sequencing analysis (Table 2) provides the peptide sequences where the proteins identified were filtered, and all high confidence matches were closely related to *B. braunii* races. Our results indicate that PSI-LHCI was present in the sucrose gradient purified fraction, further characterized by BN-PAGE and 2D native/SDS-PAGE.

**Figure 3:**
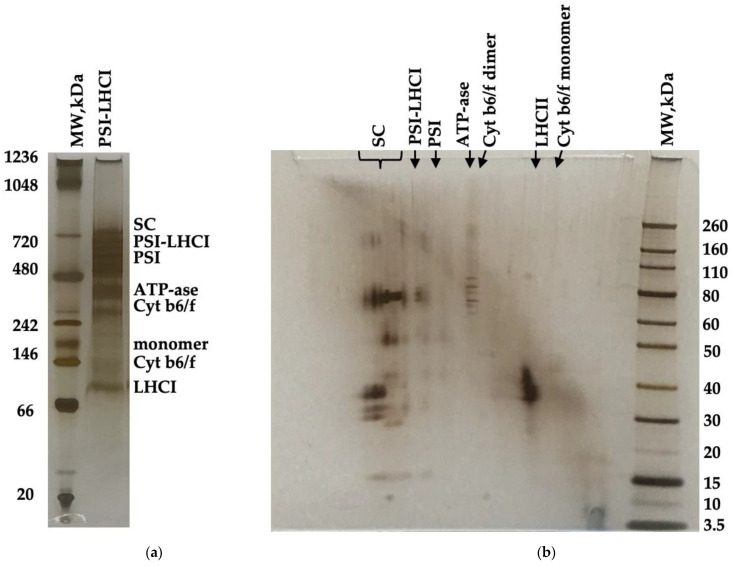
BN-PAGE separation of purified PSI-LHCI complexes from *B. braunii* at a concentration of 0.7 mg/mL Chl. The selected PSI-LHCI gel lane (**a**) was excised and applied to a two-dimensional SDS-PAGE gel to separate PSI-LHCI polypeptides and co-eluting complexes (**b**). The gels were silver-stained.

Figure 3a depicts the BN-PAGE of *B. braunii* PSI-LHCI (0.7 mg/mL Chl a). PSI-LHCI components were resolved and qualitatively visualized in a BN-PAGE gel where several bands were identified, and mass estimated using NativeMark^TM^ unstained protein standard (~20–1200 kDa). The high molecular values estimated were 669 kDa appointed to supercomplexes (SC), ~600 kDa to PSI-LHCI, 481 kDa to PSI, 324–303 kDa to ATPase, 250 kDa to dimer Cyt b6/f, 121 kDa to monomer Cyt b6/f, and 93 kDa to LHCII subunits [28,33]. Two-dimensional native/ SDS-PAGE was accomplished in a gradient 4–12% Bis-Tris gel in MES running buffer at 150 V for 1 h with the excised PSI-LHCI complex BN-PAGE gel lane obtained that underwent reduction, alkylation, and quenching, as indicated in the manufacturer’s protocol (Figure 3b). A prestained protein standard was used to estimate PSI-LHCI and co-eluting components (~3.5–260 kDa). Two-dimensional (2D) native/SDS-PAGE polypeptide spots indicated a high abundance of PSI-LHCI complex components and subunits as observed in previous reports providing an assessment of the polypeptide composition of our preparation [28,47]. Although we notice a small contribution of LHCII, a mobile antenna, judging from our 77 K fluorescence assay, it is not deemed a major contributor [34,35]. To the best of our knowledge, this is the first time an exhaustive characterization study on *B. braunii* PSI-LHCI has been conducted.

### 3.4. Cell Viability Assay in MDA-MB-231 Cells

After treatment with low critical micellar concentration detergents, such as DDM, membrane pigment–protein complexes, such as PSI-LHCI, self-assemble in larger mixed protein–detergent oblate ellipsoid micelles retaining their functional and biological properties, thus enabling interaction with the cell membrane and cytotoxic effects during in vitro studies [28]. The MTS assay monitored toxicity in TNBC MDA-MB-231 cells upon exposure to serum starvation conditions for 18 h, PSI-LHCI (6.25–50 µg/mL), and incubation times of 12 and 24 h, where *n* = 8. Untreated cells and cells incubated in extraction buffer A at the highest concentrations tested were used as a negative control and experimental control. Staurosporine was used as a positive control.

**Figure 4:**
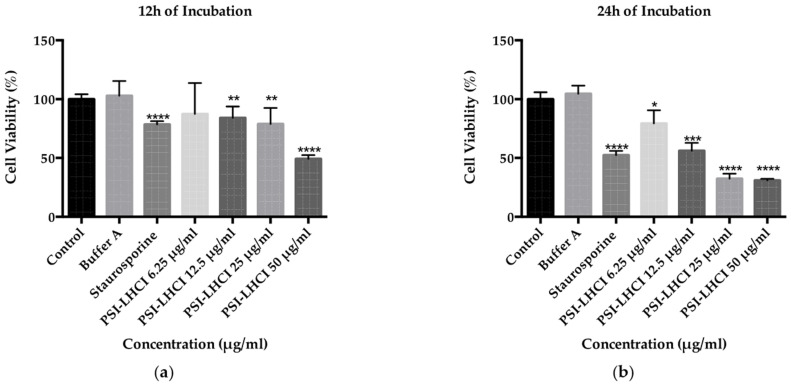
Time and concentration-dependent effect of PSI-LHCI in MDA-MB-231 cells; (**a**) effect of PSI-LHCI in cell viability following treatment with different concentrations (6.25–50 µg/mL) after 12 h of incubation. Asterisks indicate statistical significance with ** *p* < 0.01 and **** *p* < 0.0001; (**b**) effect of PSI-LHCI in cell viability following treatment with different concentrations (6.25 to 50 µg/mL) after 24 h of incubation. Incubation with the extraction buffer A at the highest concentration tested (50 µg/mL) was used as an experimental control. Staurosporine (2 µM) was used as a positive control. Untreated cells were used as a negative control. Asterisks indicate statistical significance with * *p* < 0.05, *** *p* < 0.01 and **** *p* < 0.0001. Data shown are expressed as mean ± SD performed in triplicate, where *n* = 8, and the statistical method performed used the two-way analysis of variance (ANOVA).

A decline in cell viability of 87% was observed for the 12 h incubation period, starting with the 6.25 µg/mL PSI-LHCI concentration. A reduction in viability was detected around 84% and 79% for the 12.5 and 25 µg/mL PSI-LHCI concentrations (** *p* < 0.01), respectively, and a lower value of 49% at the highest concentration tested of 50 µg/mL at a **** *p*-value of < 0.0001 (Figure 4a). After incubation with PSI-LHCI for 24 h, we observed a significant reduction in cell viability in MDA-MB-231 cells from 100% (untreated cells) to 31% with 50 µg/mL concentration (**** *p* < 0.0001), Figure 4b. At the 6.25, 12.5, and 25 µg/mL of PSI-LHCI, the time-dependent reduction in viability observed decreased from 100% to 79% (* *p* < 0.05), 56% (*** *p* < 0.01), and 32% (**** *p* < 0.0001), respectively, when compared to untreated cells. Staurosporine was used as a positive control, and the viability observed was 52% (**** *p* < 0.0001). After 24 h of incubation time of MDA-MB-231 cells with different concentrations of PSI-LHCI, the IC_50_ value attained was 14.7 µg/mL with an R-squared value of 0.9121, standard error of 1.1, and Log IC_50_ equal to 1.2.

Our MTS assay results revealed the remarkable time- and concentration-dependent effects of decreased cell viability of *B. braunii* PSI-LHCI against MDA-MB-231 cells. With an increment in PSI-LHCI concentration after 12 and 24 h of incubation, a reduction in cell viability was observed, showing an inverse relationship between proliferation and cell death. We compared our results with Staurosporine, a strong cytotoxic positive control, used at peak plasma concentration (2 µM), and negative and experimental controls. As observed in Figure 4b, after 24 h of incubation, we observed a statistically significant decrease in viable cells from 100% with negative controls to below 32% with 25 and 50 µg/mL of PSI-LHCI (**** *p* < 0.0001), in contrast to 52% with Staurosporine (**** *p* < 0.0001). We proceeded to choose the concentration point closest to the IC_50_ value (12.5 µg/mL PSI-LHCI, *** *p* < 0.01) revealed after exposure of MDA-MB-231 cells to PSI-LHCI following 24 h of incubation. Our results confirmed that PSI-LHCI induces a decrease in cell viability in the triple-negative breast cancer cell line, MDA-MB-231, with superior results than the well-known cancer cell line positive control Staurosporine [47].

### 3.5. Cellular Internalization Studies of PSI-LHCI in MDA-MB-231 Cells

The internalization of PSI-LHCI was determined by confocal laser scanning microscopy (CLSM) after MDA-MB-231 cells were seeded at 10,000 cells/well, arrested, and subsequently incubated with PSI-LHCI (12.5 μg/mL) for 24 h. After incubation and fixing, the cells were imaged using a Nikon Eclipse Ti confocal microscope. PSI-LHCI was detected at 663–738 nm after excitation at 640 nm, and DAPI was excited at 405 nm with its emission spotted from 420 to 480 nm.

**Figure 5:**
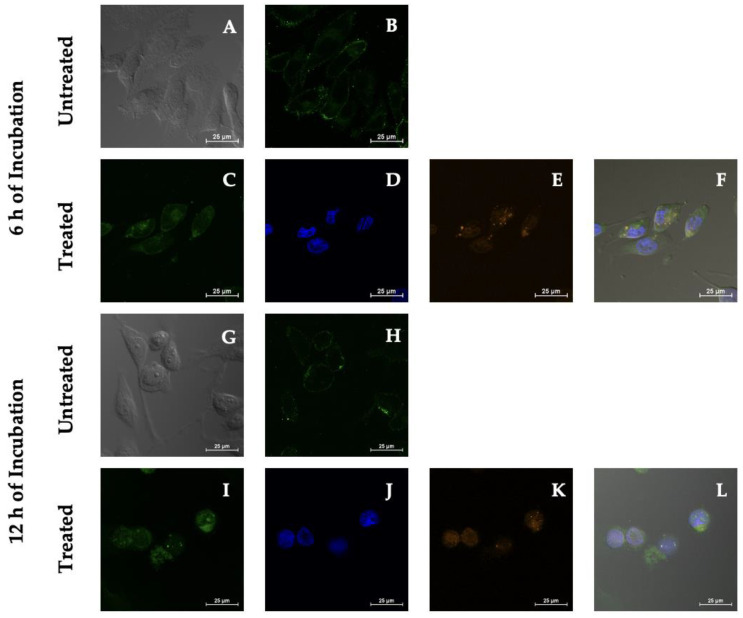
PSI-LHCI internalization in MDA-MB-231 cells by confocal microscopy studies. Phase-contrast (**A**,**G**) and green plasma membrane (**B**,**H**) of untreated MDA-MB-231 cells after 6 and 12 h of incubation. Treated MDA-MB-231 cells were incubated for 6 and 12 h with PSI-LHCI (**E**,**K**) orange in color, DAPI (**D**,**J**) blue in color, and plasma membrane (**C**,**I**) green in color. Merged pictures are presented in (**F**,**L**). All images were taken at 60× magnification. Scale bars, 25 μm.

Internalization studies after 6 and 12 h of incubation were performed and studied by monitoring the distribution of the fluorescence signal of PSI-LHCI (12.5 µg/mL) on the MDA-MB-231 cell surface and intracellular localization with CLSM, as observed in Figure 5. The MDA-MB-231 cell plasma membrane was labeled with the CellMask green plasma membrane stain to help define the cellular components bound by the cell membrane after treatment with PSI-LHCI. PSI-LHCI-treated MDA-MB-231 cells were visualized using the Z-series, a straightforward technique of analyzing multiple images at different focal planes (z-stack) of the sample of interest to determine their location within the cell [48].

Qualitative visual results of PSI-LHCI (orange), plasma membrane (green), DAPI nuclear stain (blue), and a merged image of all of the dyes provided a clear depiction of the fate of PSI-LHCI and cell membrane integrity. Green fluorescence signal intensity increased significantly compared with control from localized plasma membrane boundaries observed after 6 h of incubation to uniform cytoplasmic after 12 h (Figure 5). The mean fluorescence intensity (MFI) of MDA-MB-231 cells after 6 h of incubation with PSI-LHCI, when compared with the control, represented as mean ± standard deviation (SD), was 108.03 ± 12.23 and 94.14 ± 48.72. After 12 h of incubation with PSI-LHCI compared with the control, the MDA-MB-231 cells MFI was 503.08 ± 146.01 and 74.12 ± 10.02, respectively. Internalization and localization studies of PSI-LHCI in MDA-MB-231 cells monitored time-dependent intracellular traffic and contributed critical knowledge towards reporting non-specific internalization and PSI-LHCI damage-mediated cytotoxicity while differentiating surface membrane staining from intracellular staining, supporting the premise that PSI-LHCI does not remain on the cell’s surface; instead, it is ultimately internalized.

### 3.6. Study of ROS/Superoxide Generation in MDA-MB-231 Cells after PSI-LHCI Treatment

Cellular total ROS production and superoxide detection were directly monitored and determined using CLSM. Live MDA-MB-231 were examined in real-time after incubation with PSI-LHCI (12.5 µg/mL) for 1 or 24 h. ROS inducer (Pyocyanin 1 µM) and ROS inhibitor/scavenger (*N*-acetyl-l-cysteine) were used as positive and negative controls, respectively (Supplementary Figure S1). Sample pretreatment performed for 30 min with *N*-acetyl-l-cysteine prevented ROS generation. Compensation correction was achieved with single-stained standards.

**Figure 6:**
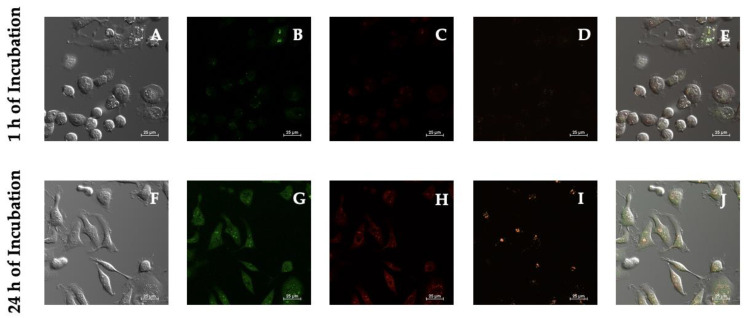
ROS/Superoxide detection microscopy studies in MDA-MB-231 live cells. Phase-contrast (**A**,**F**) of MDA-MB-231 cells after 1 and 24 h of incubation with PSI-LHCI (12.5 µg/mL). Green cytoplasmic oxidative stress signal viewed with the FITC filter (**B**,**G**); red nuclear superoxide stain visualized with the CY3 filter (**C**,**H**); orange PSI-LHCI detected with the Alexa Fluor 555 filter (**D**,**I**). Merge pictures are presented in (**E**,**J**). All images were taken at 20× magnification. Scale bars, 25 μm.

As seen in Figure 6, vivid uniform green cytoplasmic staining was observed at the occurrence of increased levels of oxidative stress and bright red nuclear staining was detected in superoxide-positive cells. Our results indicated that PSI-LHCI increased reactive oxygen species and superoxide levels following experimental conditions of 1 h versus 24 h of incubation treatment. The MFI of MDA-MB-231 cells after 1 h of incubation exhibited a PSI-LHCI (120.77 ± 5.54), oxidative stress (142.13 ± 54.24), and superoxide (98.63 ± 36.03) signal when compared to 24 h of incubation, which presented an intensified PSI-LHCI (201.45 ± 46.11), oxidative stress (366.73 ± 85.95), and superoxide (101.01 ± 37.64) signal.

Thus, a marked contrast in the fluorescence signal was observed between 1 and 24 h incubation periods for the two fluorescent dyes: green oxidative stress detection reagent and red superoxide detection reagent. Time-course fluorescence changes reinforced the constitutive internalization of PSI-LHCI after 24 h of incubation, which was monitored by the increment in orange signal in the Alexa Fluor 555 channel. An increase in signal intensity was detected over time when oxidative stress and superoxide concentration levels were monitored, surveyed in the FITC and CY3 filter channels, respectively. Hence, ROS and superoxide generation and accumulation, and a subsequent rise in oxidative stress, have thereby been further confirmed after MDA-MB-231 cells were exposed to PSI-LHCI (12.5 µg/mL) and analyzed through the ROS/Superoxide assay.

### 3.7. Annexin V/Propidium Iodide and Necrosis Assays for Cell-Death Assessment

Analyzing the unique morphological hallmarks in ongoing cell demise requires distinguishing among the different cell death pathways, including apoptosis, necrosis, and autophagy, by multiparameter studies involving qualitative and functional criteria. In our research, qualitative cell death mechanism analyses were carried out, starting with the nuclear counterstain DAPI, which can help identify the condensed nuclei of apoptotic cells. Apoptosis was studied via the Alexa Fluor 488 Annexin V and with the live and apoptotic impermeant dye propidium iodide (PI) to help distinguish apoptotic cells from dead cells, respectively. Alternatively, we used the Nuclear Green DCS1 dye to analyze dead cells. Unlike lipophilic dyes, such as FM 4–64, the fluorescent probes were selected to avoid interference with the sample, thus avoiding creating an imaging problem due to the excitation of the sample by the emission of the dye.

**Figure 7:**
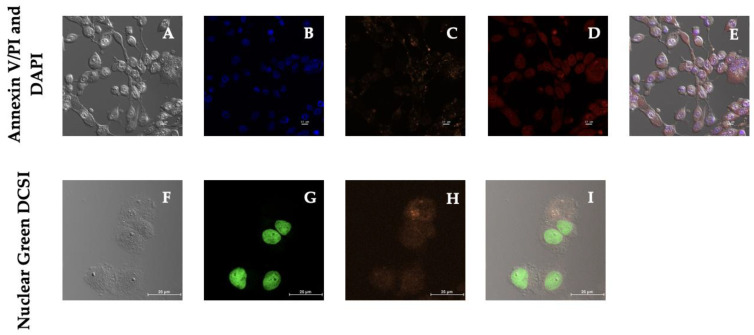
Dead cell studies after 24 h of incubation with PSI-LHCI (12.5 µg/mL). Phase-contrast images are shown in (**A**,**F**). MDA-MB-231 cells were treated with PSI-LHCI (**C**) and stained with DAPI (**B**) and PI (**D**) to determine apoptosis and PSI-LHCI localization. Merged image in magenta (**E**) represents the colocalization of DAPI (blue) and PI (red) staining and PSI-LHCI (orange) localization in MDA-MB-231 cells. Annexin V/PI and DAPI images were taken at 20× magnification. Scale bars, 10 μm. MDA-MB-231 cells were treated with orange PSI-LHCI (**H**) and stained with nuclear green DCS1 (**G**). Merged image (**I**) represents the analysis of dead cells. Nuclear Green DCS1 images were taken at 60× magnification. Scale bars, 25 μm.

An increase in ROS may cause cell death; therefore, we initially searched for hallmarks of apoptosis using DAPI, Annexin V, and PI (Figure 7). The analysis of DAPI-stained MDA-MB-231 cells unveiled a brightly stained nuclear morphology and minor condensation, initially regarded as an early apoptotic marker; however, dead or necrotic cells appeared stained bright red due to the differential uptake of the DNA binding dye PI that did not traverse the plasma membrane of apoptotic or living cells [49]. The merged image in magenta is interpreted as the overlap between neighboring fluorophores or colocalization of the blue (DAPI) and red (PI) signals and used as a discernable method to detect apoptosis; however, distinguishing apoptotic to non-apoptotic cell death is not a straightforward process [50]. After staining with Annexin V, no apparent green fluorescence was detected (Supplementary Figure S2). Therefore, this assay did not identify early apoptotic cells. Nevertheless, the stronger and condensed red fluorescence observed has been generally represented as the typical nuclear staining of dead cells [51]. Thus, an alternative cell death pathway should be responsible for the cellular cytotoxicity sustained by MDA-MB-231 cells after 24-h treatment with PSI-LHCI.

In Figure 7, we analyzed DNA content in dead cells with the DNA-selective and cell-impermeant green-fluorescent dye, Nuclear Green. MDA-MB-231 cells exhibited high-intensity fluorescence after treatment with PSI-LHCI (12.5 µg/mL) and 24 h of incubation, indicating the labeling of necrotic cells after PSI-LHCI treatment. Studies with Annexin V/PI exhibited a red concentrated cell nucleus, suggesting necrotic or late apoptosis cell staining, whereas Annexin V did not show any typical apoptosis marker. Nuclear green DCS1 results indicate MDA-MB-231 cells undergo late-stage apoptosis or necrosis after PSI-LHCI 24 h treatment. Necrosis is evidenced by the presence of dead cells, products of environmental perturbations and pathologies that led to ROS increase and mitochondria disruption, but fails to specify how the death occurred; thus, further cell death mechanism assays followed [49,52].

### 3.8. Autophagy-Associated Cell Death Assay

The final cell death mechanism examined was ROS-induced autophagy, also known as macroautophagy via the LC3 lysosomal pathway. The housekeeping ROS-induced autophagic cell death pathway was determined following treatment with PSI-LHCI (12.5 µg/mL) in synchronously nutrient-starved MDA-MB-231 cells. During the highly regulated lysosome-based cellular recycling process, degradation of cytoplasmic components is mediated by the double-membrane vesicles or autophagosomes, the morphological hallmark of macroautophagy [53].

The autophagy inducer rapamycin, the lysosomal activity inhibitor, and autophagic flux detector chloroquine were used as positive controls (Supplementary Figure S3). Untreated MDA-MB-231 cells were sampled in complete media and under serum-starved conditions. Complete growth media and serum starvation conditions were used to measure autophagic vacuoles and monitor autophagic flux directly. Fluorescence intensity was detected in autophagic vacuoles after incubation with the green microscopy dual detection reagent by confocal microscopy. Fixed-stained cells were monitored for autophagy activity at 40× magnification using the standard FITC filter set for imaging the autophagic signal. In contrast, the nuclear signal was obtained using the DAPI channel.

**Figure 8:**
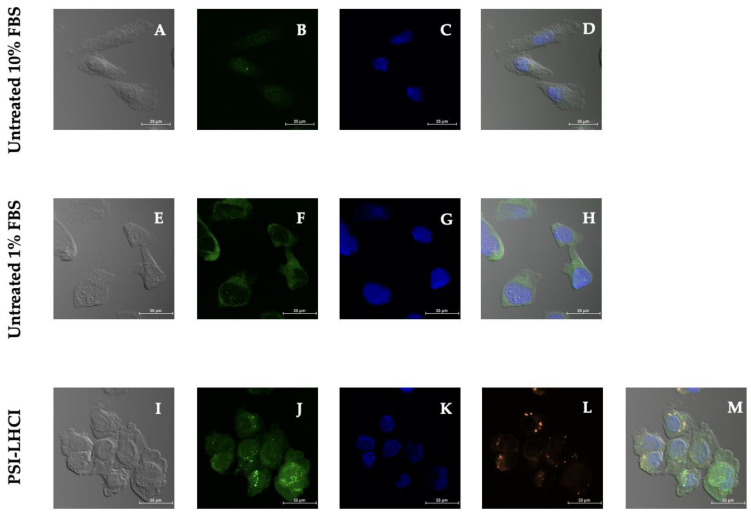
Autophagy confocal microscopy studies in MDA-MB-231 cells. Phase-contrast (**A**,**E**,**I**) and merged images (**D**,**H**,**M**) of MDA-MB-231 cells, untreated cells tested in 10% FBS, 1% FBS, and cells treated with PSI-LHCI (12.5 µg/mL), respectively. Blue DAPI nuclear stain (**C**) and green autophagic vesicles (**B**) of cells in complete medium. Blue DAPI nuclear stain (**G**) and green cytoplasm autophagic signal (**F**) of cells in a serum-deprived medium. MDA-MB-231 cells after 24 h of incubation with PSI-LHCI in a serum-deprived medium. Green autophagic vesicles (**J**), blue DAPI nuclear stain (**K**), and PSI-LHCI (**L**) after treatment. All images were taken at 40× magnification. Scale bars, 25 μm.

The accumulation of the green detection reagent, represented by a uniform but diffused positive autophagy signal in the cytoplasm, was identified in serum-starved untreated cells. Localized globular autophagic vacuoles or vesicles, identified as dotted structures of increased fluorescence intensity, and a dispersed autophagy signal in the cytoplasm and nucleus, were detected in PSI-LHCI-treated MDA-MB-231 cells incubated in L-15 medium with 1% FBS (Figure 8). The starvation medium is recognized as an autophagy inducer treatment.

Experimental treatment of MDA-MB-231 cells with PSI-LHCI after 24 h of incubation time revealed a bright fluorescent orange signal obtained using the Alexa Fluor 555 channel corresponding to the auto-fluorescent pigment–protein complex. The blue nuclear stain Hoechst exhibited typical nuclear staining. The green detection signal observed in MDA-MB-231 cells as punctuate structures in the autophagic vacuoles and diffused throughout the nucleus and cytoplasm is presumably the LC3 autophagosome marker protein. These results confirm that the ROS-rich PSI-LHCI pigment–protein complex in serum starvation conditions induces autophagy [54]. To date, as far as we know, this is the first study to report the biological effects and mechanisms of cell death induced by *B. braunii* PSI-LHCI against the TNBC cell line MDA-MB-231.

A non-toxic targeted *Spinacia oleracea* PSI delivery formulation to cancer cells has been investigated in vivo in the C57BL/6J female mice model and significant tumor growth inhibition after treatment was observed [11]. However, strategies in designing microalgal PSI-LHCI delivery systems to ensure the specificity, stability, efficacy, and increased bioavailability of the pigment–protein complex for in vivo application are critical for forthcoming studies.

## 4. Conclusions

In summary, a discontinuous sucrose density gradient ultracentrifugation technique was utilized as a purification process to generate PSI-LHCI from *Botryococcus braunii* UTEX2441. Characterization results were evaluated and confirmed by absorbance and fluorescence spectroscopic studies, CD measurements, pigment, and polypeptide composition analysis. This manuscript highlights the PSI core complex and surrounding LHCI antenna system organization, furthering the understanding and studies of this organism’s PSI. *B. braunii* is a green microalga highly studied due to its oil generation and use in the biofuel industry. However, its biomedical application examined in this study sets the groundwork for broadening PSI’s relevance and use. Exposure of MDA-MB-231 cells to PSI-LHCI showed cell death following autophagic and necrotic cell death mechanisms. This study explores the potential of *B. braunii* PSI-LHCI biological applications; nevertheless, it requires further evaluation and optimization delivery technologies.

## Figures and Tables

**Table 1:** Pigment content of *B. braunii* cells, thylakoid membrane, and PSI-LHCI complex extracted in 90% methanol.

Parameter	Cells	Thylakoid Membrane	PSI-LHCI
Carotenoids (μg/mL)	2.1 ± 0.4	0.28 ± 0.02	0.31 ± 0.01
Chl *a* (μg/mL)	1.6 ± 0.5	1.02 ± 0.03	1.50 ± 0.01
Chl *b* (μg/mL)	0.8 ± 0.3	0.53 ± 0.09	0.45 ± 0.03
Chl *a*/*b* ratio (*w*/*w*)	1.93± 0.06	2.0 ± 0.3	3.4 ± 0.2
Total Chl (μg/mL)	2.4 ± 0.8	1.6 ± 0.1	1.94 ± 0.04

Average values are presented as means of two independent experiments ± SD.

**Table 2:** Polypeptide composition of *B. braunii* PSI-LHCI complex. Proteins were identified by LC-MS/MS.

Protein Identification	Accession Number	Gene	De Novo Sequence Protein Fragment	Total Score
Photosystem I P700 chlorophyll *a* apoprotein A1	A0A097KQ63	psaA	DYDPTNNYNNLLDR ALSITQGR FPCDGPGR VAPAIQPR LLDAGIDPK	49.90
Photosystem I P700 chlorophyll *a* apoprotein A2	A0A097KQ46	psaB	DFGYSFPCDGPGR	8.56
Photosystem I iron-sulfur center	A0A097KQ83	psaC	IYDTCIGCTQCVR VYLGAETTR	16.28
ATP synthase subunit beta, chloroplastic	A0A097KQ64	atpB	VALVYGQMNEPPGAR VALVYGQMNEPPGAR TVLIMELINNIAK VVDLLAPYR IGLFGGAGVGK LSIFETGIK	61.09
ATP synthase subunit alpha, chloroplastic	A0A097KQ55	atpA	TAVAIDTILNQK ELIIGDRQTGK ELIIGDR	24.99
ATP synthase subunit alpha	A0A0U2F033	atp1	ELIIGDRQTGK AVDSLVPIGR ELIIGDR STVAQLVK VLDTLGQPIDGK	22.08
Cytochrome f	A0A097KQ58	petA	VQLAEMNF	2.21

## Data Availability

Data available upon reasonable request.

## Supplementary Materials

The following supporting information can be downloaded at: https://www.mdpi.com/article/10.3390/biotech11020009/s1, Figure S1: ROS/Superoxide detection microscopy studies in MDA-MB-231 live cells. Phase-contrast MDA-MB-231 untreated cells, positive (Pyocyanin) and negative control ROS inhibitor (*N*-acetyl-l-cysteine). Green cytoplasmic stain (FITC filter) and red nuclear stain (CY3 filter). All images were taken at 20× magnification. Scale bars, 25 μm, Figure S2: Confocal microscopy studies in the MDA-MB-231 cell line. Cells were treated with PSI-LHCI (12.5 µg/mL) after 24 h of incubation to determine apoptosis using Annexin V. Image was taken at 20× magnification. Scale bar, 10 μm, Figure S3: Autophagy confocal microscopy studies in control MDA-MB-231 cells. Phase-contrast of MDA-MB-231 cells, untreated cells, autophagy inducer rapamycin, and chloroquine as a positive control. Blue nuclear stain (DAPI filter) and green autophagic vesicles (FITC filter) of cells in complete medium. All images were taken at 40× magnification. Scale bars, 25 μm.
