# Supplementary material for: Oxidative Stress- and Autophagy-Inducing Effects of PSI-LHCI from Botryococcus braunii in Breast Cancer Cells
Source: BioTech (Basel). 2022 Mar 31;11(2):9. doi: 10.3390/biotech11020009 (PMC9264392; doi:10.3390/biotech11020009)
Supplement: Supplementary file 1 [file biotech-11-00009-s001.zip › biotech-1599419-supplementary.pdf]

Supplementary Materials for

Oxidative Stress- and Autophagy-Inducing Effects of PSI-LHCI from *Botryococcus braunii* in Breast Cancer Cells

Freisa M. Joaquín-Ovalle [1], Grace Guihurt [1], Vanessa Barcelo-Bovea [1], Andraous Hani-Saba [1], Nicole C. Fontanet-Gómez [1], Josell Ramirez-Paz [1], Yasuhiro Kashino [2], Zally Torres-Martinez [1], Katerina Doble-Cacho [1], Louis J. Delinois [1], Yamixa Delgado [3,*], and Kai Griebenow [1,*]

[1] Department of Chemistry, University of Puerto Rico, Río Piedras Campus, San Juan 00925, Puerto Rico; freisa.joaquinovalle@upr.edu (F.M.J.O.); gracegpr@gmail.com (G.G.); vanessabarcelo1@gmail.com (V.B.B.); andraous.saba@upr.edu (A.H.S.); nicole.fontanet@upr.edu (N.C.F.G.); josellyaima@hotmail.com (J.R.P.); zallytorres@gmail.com (Z.T.M.); katerina.doble@edu.uag.mx (K.D.C.); delinoisjeanlouis@gmail.com (L.J.D.)

[2] Graduate School of Science, University of Hyogo, Hyogo 678-1297, Japan; kashino@sci.u-hyogo.ac.jp

[3] Biochemistry & Pharmacology Department; San Juan Bautista School of Medicine, Caguas 00725, Puerto Rico; ydelgado@sanjuanbautista.edu

* Correspondence: ydelgado@sanjuanbautista.edu, kai.griebenow@gmail.com

Supplementary Figures

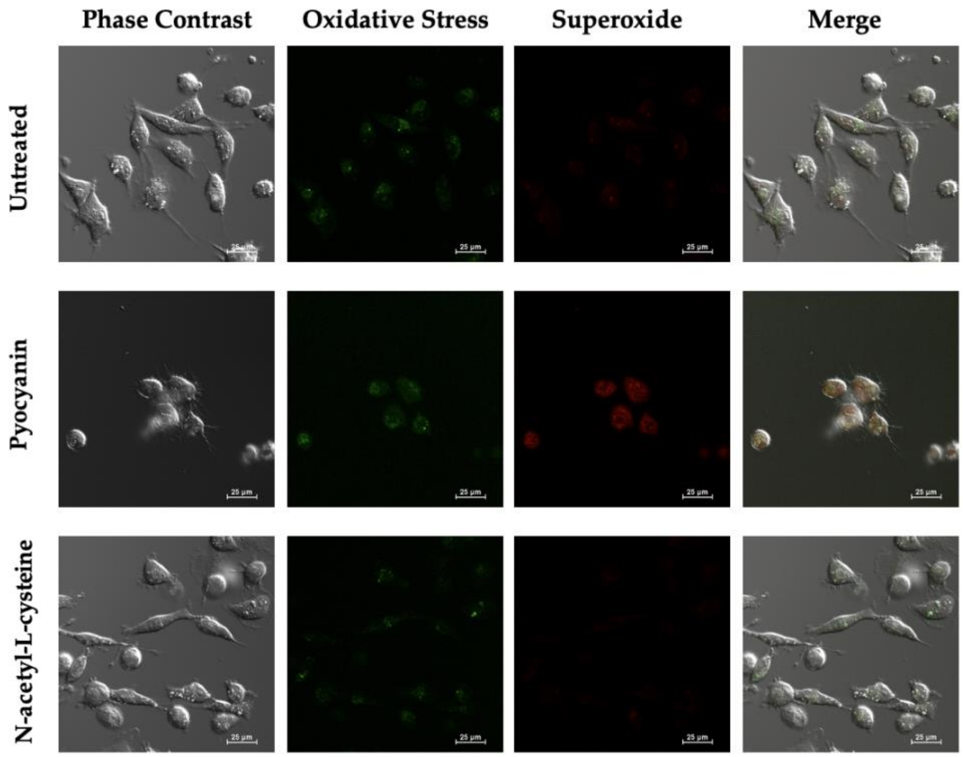

Figure S1. ROS/Superoxide detection microscopy studies in MDA-MB-231 live cells. Phase-contrast MDA-MB-231 untreated cells, positive control ROS inducer (Pyocyanin), and negative control ROS inhibitor (N-acetyl-L-cysteine). Serum-starved conditions increased the green oxidative stress signal. Red nuclear and

cytoplasmic staining was detected in superoxide-positive cells but not in negative control cells. Green cytoplasmic stain (FITC filter) and red nuclear stain (CY3 filter). Images were taken at 20× magnification. Scale bars, 25 μm.

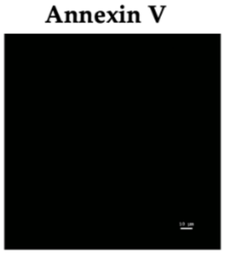

Figure S2. Confocal microscopy studies in the MDA-MB-231 cell line. Cells were treated with PSI-LHCI (12.5 μg/mL) after 24 h of incubation to determine cell death using Annexin V Alexa Fluor excited at 488 nm; early apoptosis was not detected. Image was taken at 20× magnification. Scale bar, 10 μm.

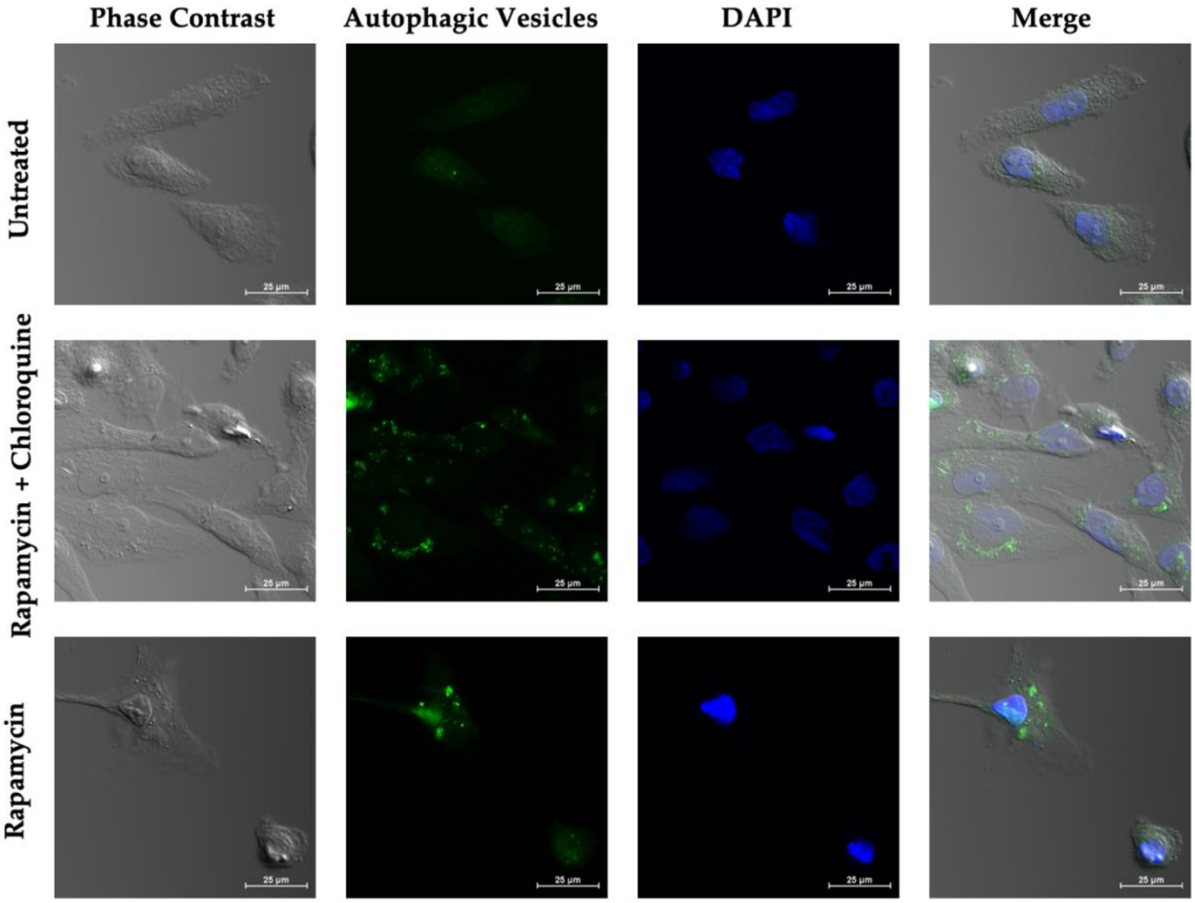

Figure S3. Autophagy confocal microscopy studies in control MDA-MB-231 cells. Phase-contrast of MDA-MB-231 cells, untreated cells, autophagy inducer rapamycin, and chloroquine as a positive control. The accumulation of the green detection reagent, represented by localized globular autophagic vacuoles or

vesicles, was identified in the positive controls, rapamycin, rapamycin & chloroquine as dotted structures of increased fluorescence intensity. Blue nuclear stain (DAPI filter) and green autophagic vesicles (FITC filter) of cells in complete medium. Images were taken at 40× magnification. Scale bars, 25 μm.
